# Expression of virus-like particles (VLPs) of foot-and-mouth disease virus (FMDV) using *Saccharomyces cerevisiae*

**DOI:** 10.1007/s00253-023-12902-9

**Published:** 2024-01-09

**Authors:** Ngoc My Tieu Le, Kum-Kang So, Jeesun Chun, Dae-Hyuk Kim

**Affiliations:** 1 Department of Bioactive Material Sciences, Jeonbuk National University, Jeonju, 54896 Jeollabuk-do Republic of Korea; 2 Institute for Molecular Biology and Genetics, Department of Molecular Biology, Jeonbuk National University, Jeonju, Jeollabuk-Do Republic of Korea

**Keywords:** Virus-like particle, FMDV, *Saccharomyces cerevisiae*, Ribosomal skipping, Capsid protein

## Abstract

**Abstract:**

We engineered *Saccharomyces cerevisiae* to express structural proteins of foot-and-mouth disease virus (FMDV) and produce virus-like particles (VLPs). The gene, which encodes four structural capsid proteins (VP0 (VP4 and VP2), VP3, and VP1), followed by a translational “ribosomal skipping” sequence consisting of 2A and protease 3C, was codon-optimized and chemically synthesized. The cloned gene was used to transform *S. cerevisiae* 2805 strain. Western blot analysis revealed that the polyprotein consisting of VP0, VP3, and VP1 was processed into the discrete capsid proteins. Western blot analysis of 3C confirmed the presence of discrete 3C protein, suggesting that the 2A sequence functioned as a “ribosomal skipping” signal in the yeast for an internal re-initiation of 3C translation from a monocistronic transcript, thereby indicating polyprotein processing by the discrete 3C protease. Moreover, a band corresponding to only VP2, which was known to be non-enzymatically processed from VP0 to both VP4 and VP2 during viral assembly, further validated the assembly of processed capsid proteins into VLPs. Electron microscopy showed the presence of the characteristic icosahedral VLPs. Our results clearly demonstrate that *S. cerevisiae* processes the viral structural polyprotein using a viral 3C protease and the resulting viral capsid subunits are assembled into virion particles.

**Key points:**

*• Ribosomal skipping by self-cleaving FMDV peptide in S. cerevisiae.*

*• Proteolytic processing of a structural polyprotein from a monocistronic transcript.*

*• Assembly of the processed viral capsid proteins into a virus-like particle.*

**Supplementary Information:**

The online version contains supplementary material available at 10.1007/s00253-023-12902-9.

## Introduction

Foot-and-mouth disease (FMD) is a highly contagious disease caused by infection with FMD virus (FMDV) that is capable of infecting more than 70 species of cloven-hoofed animals (Fenner et al. 1993). As of 2013, more than 100 countries were engaged in the search for an efficient means of controlling FMD (Jamal and Belsham 2013). FMDV contains a single-stranded positive-sense RNA genome of 8.5 kb, that belongs to the genus *Aphthovirus* of the family *Picornaviridae*, in which a single-stranded RNA viral genome encodes a single polyprotein, which is subsequently cleaved into four structural proteins (VP1–VP4), 10 non-structural proteins (L^pro^, 2A, 2B, 2C, 3A, 3B_1–3_, 3C^pro^, and 3D^pol^), along with some cleavage intermediate forms. After an initial translation, viral precursor P1 peptide is processed into VP0, VP1, and VP3 via the activity 3C protease, which arises from precursor P3 non-structural polypeptide. After maturation, capsid protein precursor VP0 undergoes further self-cleavage into capsid proteins VP2 and VP4. VP1, VP2, and VP3 are located at the surface of the virus, while VP4 is buried inside the capsid. The surface of an FMDV particle is smooth and spherical, and possesses a size of approximately 30 nm in diameter without the envelope. FMDV has a *T* = pseudo3 icosahedral structure made of 60 tightly packed asymmetrical protomers. FMDV’s structural proteins are responsible for assembling viral capsids, maintaining stability of viruses, binding to host cells, determining antigen binding specificity, and play a vital role in the process of viral recognition and infection (Domingo et al. 2003; Fry et al. 1999; Jamal and Belsham 2013). Due to the high mutation ratio and rapid proliferation of FMDV, the virus has been divided into seven main serotypes (A, O, C, Asia1, South African Territories (SAT)1, SAT2, and SAT3), which are distributed non-uniformly around the world. Furthermore, numerous subtypes and variants from each main serotype have been evolved. Among these, the serotypes O, A, and C are most common and responsible for outbreaks in Europe, America, Asia, and Africa (Jamal and Belsham 2013). There is no cross-reactive immune response between the seven serotypes, and only a partial cross-protection between the different subtypes of the same serotypes exists (Robinson et al. 2016). Considering the antigenic diversity among serotypes, the control and prevention of FMD via vaccine development requires further consideration (Jamal and Belsham 2013).

Commercially available FMD vaccines consist of purified inactivated whole virus preparations, but various drawbacks associated with these including quality control and residual infectivity have prompted the development of recombinant vaccine alternatives. Empty capsid vaccines, such as virus-like particles (VLPs), while more complicated than simple subunit vaccines, are a fascinating option, since they preserve the epitope structures without employing viruses during the vaccine manufacturing process (Rodriguez and Grubman 2009). VLPs are self-assembling and arranged in a certain order by multiple copies of structural proteins of a virus, which is similar to those of nature virus particles (Dong et al. 2014). VLPs consist of the entire collection of immunogenic sites on virus particles without the infectious genetic material which is necessary for the synthesis, processing, and assembly of structural proteins into empty viral capsids (Grubman et al. 1985; Li et al. 2008). Although VLPs can be spontaneously generated in vitro during culture of cells, they can be produced by heterologous expression of capsid subunits, followed by self-assembly. A variety of viral gene expression using heterologous systems, including bacteria, baculovirus, insect larvae, mammalian cells, and plants, has been investigated as a means of creating recombinant FMDV VLPs (Bhat et al. 2013; Cao et al. 2009; Kumar et al. 2016; Kushnir et al. 2012; Veerapen et al. 2018). The formation of recombinant FMDV VLPs requires the expression of virus structural proteins VP0, VP3, and VP1 (Jamal and Belsham 2013), which can be obtained by expressing either the individual structural proteins or the polyproteins comprising the P1-2A precursor and the 3C protease (Belsham and Botner 2015). In general, the latter has proven more efficient than the former. Accordingly, we tested whether VLP formation was possible by expressing the polyprotein of P1-2A-3C using yeast.

Baker’s yeast *Saccharomyces cerevisiae* has been an attractive experimental system for basic research because of their great advances such as the huge amount of currently available genetic, molecular, and cellular information (Blandin et al. 2000; Brachat et al. 2003; Cliften et al. 2001; Dunham et al. 2002; Garfinkel 2005; Goffeau et al. 1996, 1997), and the existence of well-known protocols for the genetic manipulation of laboratory strains (Ausubel et al. 1998; Romanosa et al. 1992). *S. cerevisiae* also possesses the advantages of a single cell microorganism, gene expression at a high level, ease of cultivation and scale-up capacity, and post-translational modifications and secretion. In fact, within the area of biotechnology, it is the most popular eukaryotic host organism for heterologous expression (Waegeman and Soetaert 2011). *S. cerevisiae* has been considered as a generally recognized as safe (GRAS) organism, which generates its recombinant proteins and development processes readily applicable with no further consideration. Furthermore, due to the strong adjuvant property of yeast derivatives, yeast expression offers an attractive system for vaccine production and development (Bal et al. 2018a,b; Cen et al. 2021; Gao et al. 2021; Goh et al. 2021; Kim et al. 2010; Seif et al. 2016; So et al. 2021). Some of our recent studies have demonstrated that the dengue viral epitope expressed by this yeast expression induced a well-established immune response to dengue virus (Bal et al. 2018a,b; Kim et al. 2010; Nguyen et al. 2013, 2015; Seif et al. 2016). Moreover, the cell wall of yeast can act as a capsule for expressed antigens when orally delivered to the body, negating the purification step and providing protection from digestion. Therefore, the potential of mucosal vaccine development is more readily apparent than other systems previously used to produce FMD VLP. In this study, we engineered *S. cerevisiae* to produce FMDV VLPs by expressing the polyprotein of P1-2A-3C. We tested whether the polyprotein precursor of structural FMDV protein P1 is processed into capsid subunits by the FMDV 3C protease (which is known to be independently translated by a translational “ribosomal skipping” signal peptide 2A (Atkins et al. 2007; Donnelly et al. 2001) and the resulting capsid proteins assembled into VLPs. To our knowledge, this is the first report to produce FMDV VLPs using recombinant *S. cerevisiae*.

## Materials and methods

### Strains and culture conditions

*Escherichia coli* TOP10 (Thermo Fisher Scientific, Waltham, MA, USA) was generally used to clone genes. *E. coli* BL21 (Qiagen, Hilden, Germany) and M15 (Qiagen) strains were used to prepare the purified antigen for each viral capsid protein. *S. cerevisiae* 2805 (*MATa pep4::HIS3 prb1-Δcan1 GAL2 his3 ura3-52*) was the yeast strain used for heterologous expression. Yeast and bacterial strains were cultured, as previously described (So et al. 2021).

### Construction of yeast expression vectors and transformation

**Fig. 1:**
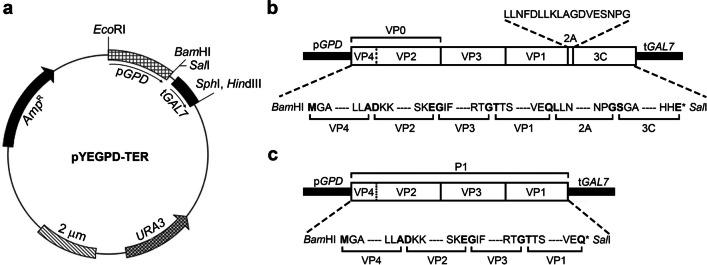
**a** Schematic map of the yeast episomal pYEGPD-TER expression vector, showing 2-µm origin of replication, *URA3* and *Amp*.^R^ markers, and **b** the amino acid sequence around the junction sites including the promoter/start codon of P1, P1/2A, and 2A/3C in the pYEGPD-P1-2A-3C construct, and **c** the amino acid sequence of P1 in the pYEGPD-P1 construct (shown in bold)

Fusion gene construct (P1-2A-3C) consisting of polyprotein precursor P1, 2A, and protease 3C of FMDV serotype O (GenBank accession no. AY593823.1) was chemically synthesized (Bioneer Corporation, Daejeon, Korea) after codon optimization for yeast expression (GenBank accession no. OR283030). Restriction enzyme sites such as *Bam*HI and *Sal*I were introduced on 5′ and 3′ ends of the synthetic gene by PCR (Supplemental Table S1), and PCR amplicons were subsequently cloned into the pGEM T-easy vector (Promega, Madison, WI, USA). The fusion construct was inserted into *Bam*HI and *Sal*I-digested yeast episomal plasmid pYEGPD-TER (Mo et al. 2005), which carries sequences from the genes for glyceraldehyde-3-phosphate dehydrogenase (*GPD*) and galactose-1-phosphate uridyl transferase (*GAL7*) as promoter and terminator, respectively, to construct pYEGPD-P1-2A-3C (Fig. 1). The P1 gene alone (without 2A and 3C (P1)) was similarly engineered to create a recombinant vector pYEGPD-P1 as a control. The lithium acetate transformation (Gietz et al. 1992) of *S. cerevisiae* 2805 with the vectors carrying the P1-2A-3C gene and the P1 gene was performed. Yeast cells transformed with empty pYEGPD-TER vector were constructed as a negative control. The presence of the transformed recombinant plasmid was assessed by colony PCR analysis and *E. coli* back-transformation (So et al. 2021).

All primers used to clone the *S. cerevisiae* expression cassettes are provided in Supplemental Table S1.

### Northern blot analysis

RNA extraction was performed as previously reported (Lim et al. 2003) and quantified using a Multiskan Go UV spectrophotometer (Thermo Fisher Scientific). About 30 μg of RNA was electrophoretic separated through 1.2% formaldehyde-containing agarose gel and transferred to an Amersham Hybond™ membrane (Cytiva, Marlborough, MA, USA). Radioactively labeled probe with α-[^32^P] was used to hybridize the RNA blot using modified Church buffer (250 mM Na_2_HPO_4_; 1 mM EDTA; 7% SDS; 0.17% H_3_PO_4_; 1% hydrolysated casein).

### Western blot analysis

FMDV antigen expression and purification were performed using *E. coli* expression of viral capsid proteins (VP1, VP2, and VP3) and 3C protein. The gene encoding VP1 was cloned into the pCold II vector (Takara Bio Inc., Shiga, Japan) and the genes of VP2, VP3, and 3C were cloned into the pQE-30 vector (Qiagen). The resulting expression recombinant plasmids were then transformed into *E. coli* expression hosts (BL21 for pCold II and M15 for pQE-30). Ni–NTA agarose column (Invitrogen, Waltham, MA, USA) was used to purify *E. coli*-expressed proteins in 8-M urea buffer. SDS-PAGE with anti-His-tag antibody was used to analyze for purity of each antigen. Anti-FMDV antibodies were obtained by injecting purified VP1, VP2, VP3, and 3C proteins into 8-week-old BALB/c mice (Charles River Laboratory, Wilmington, MA, USA) with complete and incomplete Freund’s adjuvant. At 4 days after the third booster induction, antisera samples were taken.

Protein samples from yeast transformants were prepared, as previously described (Mo et al. 2005). About 150 μg of total proteins were separated by 12% SDS-PAGE gel and blotted onto a nitrocellulose membrane. The target proteins (VP1, VP2, VP3, and 3C) were detected using corresponding (anti-VP1, -VP2, -VP3, and -3C) antibodies. Goat anti-mouse IgG conjugated to alkaline phosphatase was used as a secondary antibody (Sigma-Aldrich, Burlington, MA, USA). The color detection was performed by BCIP/NBT in TMN buffer (100 mM Tris, pH 9.5; 5 mM MgCl_2_; 100 mM NaCl).

### Observation of VLP using EM

Bead-treated yeast cell lysates were centrifuged for removal of cell debris. The viral particles were concentrated by ultracentrifugation at 119,000 × g for 2 h at 4 °C. The resulting pellets were resuspended in 0.1 M sodium phosphate buffer, loaded onto 10 to 50% sucrose gradient layers, and further centrifuged at 70,000 × g for 4 h at 4 °C. The collected fractions were dialyzed in PBS and analyzed by SDS-PAGE and western blot analysis. The structure of the virus particles was visualized on an H-7650 (Hitachi, Tokyo, Japan) transmission electron microscope (TEM) using negative staining with a 2% solution of uranyl acetate.

### Immune response of expressed VLPs

The antigenicity of the yeast-expressed VLPs was tested with a commercially available serotype-specific FMDV antigen detection kit, VDRG® FMDV 3Diff/PAN Ag Rapid Kit (Median Diagnostics Inc., Chuncheon, Korea), according to the manufacturer’s instructions.

## Results

### Construction of the VLP expression vector for *S. cerevisiae* transformation

Yeast episomal expression vector pYEPGPD-TER, which has been used to express various heterologous proteins in *S. cerevisiae* (Bal et al. 2018a,b; Kim et al. 2011; Lim et al. 2003; Nguyen et al. 2013; Shin et al. 2001), was used for expression of the target genes. This vector contains an expression cassette under the control of constitutive *GPD* promoter, the *GAL7* terminator, and a 2-µm-yeast origin of replication sequence that allows it to replicate independent of chromosomal DNA for a high copy number vector. The DNA sequence for the fusion construct of a precursor protein of P1-2A and 3C (P1-2A-3C) from a Turkey strain of FMD serotype O virus (O1/Manisa/TUR/69, GenBank accession no. AY593823.1) was chemically synthesized as a single gene construct after the codon-optimization for yeast expression and cloned into the pGEM T-Easy Vector, the result of which will hereafter be referred as pP1-2A-3C. In order for the directional cloning of the expression cassette into expression vector pYEPGPD-TER, *Bam*HI and *Sal*I restriction sites were added to the P1-2A-3C gene during the PCR amplification. Sequencing validated that the resulting PCR amplicon of 2.9 kb was ligated into the pGEM T-easy vector. The *Bam*HI and *Sal*I-digested pYEPGPD-TER vector was ligated to the *Bam*HI and *Sal*I-digested P1-2A-3C, and the resulting recombinant vector (pYEPGPD-P1-2A-3C) for yeast expression (Fig. 1) was verified by sequencing and used for further transformation of *S. cerevisiae*.

### Analysis of *S. cerevisiae* transformation

Twenty colonies of putative transformants were chosen on uracil-deficient selective medium. PCR using colonies as the template DNA source was performed to confirm the presence of pYEGPD-P1-2A-3C in the yeast. The PCR results showed that the presence of desired 640-bp PCR segment was amplified in all colonies by using primers for amplification of 3C sequence (Supplemental Fig. S1). In addition, transformation of *E. coli* using the preparation of plasmid DNAs from those yeast transformants, followed by restriction enzyme analysis of the plasmid DNAs, verified the presence of corresponding recombinant plasmid pYEGPD-P1-2A-3C in the yeast cells.

The accumulation of target gene transcripts from selected transformants was measured using 3-day cultured cells. Northern blotting showed successful transcription of P1-2A-3C in all 13 selected transformed clones (data not shown). Variations in the transcription level of the P1-2A-3C gene were observed among the transformants, attributed to discrepancy in plasmid copy number (Lim et al. 2003). Thus, seven transformants (TpYEGPD-P1-2A-3C-2, -3, -4, -5, -6, -7, and -11) showing high transcription levels were selected for subsequent analysis. A growth curve for each of these selected transformants was measured over 5 days. There was no significant difference detected among transformants and, even more tellingly, a similar number of cells (i.e., 3.0–4.0 × 10^11^ cells per liter) were observed at the stationary phase reached after a 3-day culture (Lim et al. 2003; Shin et al. 2001). These results indicate that growth abnormalities did not exist due to the recombinant protein P1-2A-3C in the selected transformants (data not shown).

The temporal pattern of P1-2A-3C expression was analyzed by northern blotting of TpYEGPD-P1-2A-3C-3, which was selected from among the seven transformants as representative. As shown in Supplemental Fig. S2, the transcript accumulation peaked after 48 h of cultivation and then gradually decreased. In contrast, the *GPD* transcription as an internal control showed a high expression up to the late stationary phase of 5 days of cultivation.

### Expression and processing analysis of the protein product of the target gene

**Fig. 2:**
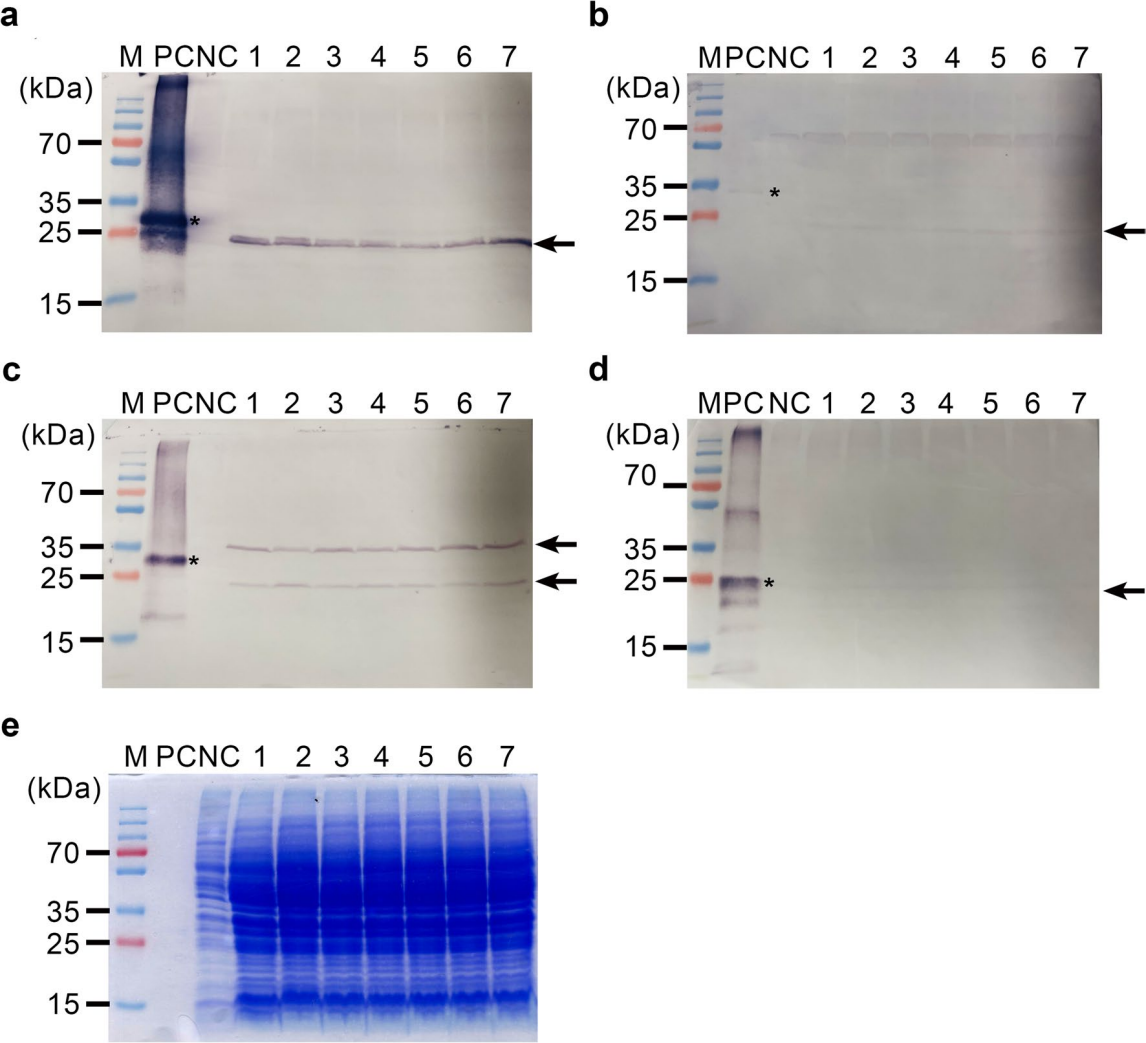
Expression analysis of yeast codon optimized P1-2A-3C polyprotein. Western blot analysis of yeast expressing P1-2A-3C polyprotein using antibodies against VP3 (**a**), VP1 (**b**), VP2 (**c**), and 3C (**d**). Lanes 1–7 contain protein preparations from seven selected yeast transformants cultured for 3 days at 30 °C. *E. coli*-expressed corresponding capsid proteins (VP1, VP2, and VP3), and 3C protease are used as PC. NC contains the protein preparation from mock (vector only) transformant. **e** A representative twin SDS-PAGE gel stained with Coomassie blue is shown as a comparison. Asterisks and arrows indicate the corresponding capsid proteins from *E. coli* and from yeast, respectively

Cell free extracts from the selected seven strains were evaluated by western blot analysis for the expression of the protein product of the P1-2A-3C gene. Using the anti-VP3 antibody, a distinctive antibody-reacting band was observed at 24 kDa, its size corresponding to that of the estimated size of VP3 capsid protein (Fig. 2a). The use of anti-VP1 antibody resulted in the detection of a cross-reacting protein band at 24 kDa, corresponding to the estimated size of VP1 capsid protein (Fig. 2b), although this band was not as strong as that associated with the use of the anti-VP3 antibody. The anti-VP2 antibody cross-reacted to two distinctive protein bands at 37 and 24 kDa (Fig. 2c); as the intermediate viral capsid precursor VP0 comprises VP4 and VP2, the cross-reacting 37 and 24 kDa bands, respectively, represented VP0 (VP4–VP2) and VP2 capsid proteins. In addition, the anti-3C antibody detected a cross-reacting protein band at 23 kDa, although its very low intensity suggests a minimal expression of the 3C protein (Fig. 2d).

### Electron microscopy of VLPs

**Fig. 3:**
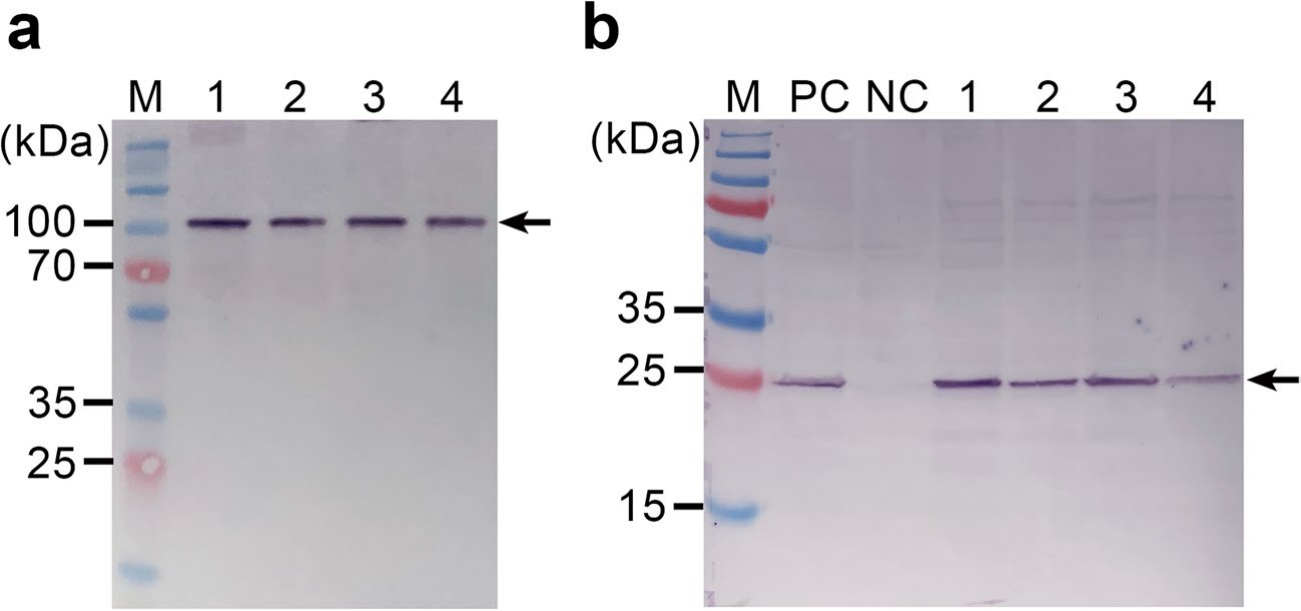
Western blot analysis of partially purified VLPs. Western blot analysis using anti-VP3 antibody was performed with and without boiling treatment of the protein samples prior to gel loading. **a** contains protein samples without boiling, and **b** are samples treated with boiling prior to loading the gel. Lanes 1–4 contain samples from the selected fractions. PC and NC contain the protein preparations from yeast expressing P1-2A-3C polyprotein and mock (vector only) transformant, respectively. Arrows indicate the corresponding capsid proteins of VLPs

Partial purification of VLPs in yeast cells was conducted by sucrose density gradient ultracentrifugation. Two distinct bands were observed in the middle part of the sucrose gradient after ultracentrifugation, subsequent to which sample fractions containing 200 μl were collected. Western blot analysis was performed using fractions obtained from ultracentrifugation prior to electron microscopy (EM) observation. As shown in Fig. 3, when samples from each fraction were not boiled prior to gel loading, they showed a large single antibody-reacting band above 100 kDa, corresponding to immature protomers of FMDV. However, boiled samples revealed an antibody-reacting band corresponding to the expected size of the capsid subunit. These results suggest that the band above 100 kDa is VLP-originated immature protomers and not unprocessed P1-2A-3C. These results were consistent with a previous finding that FMDV capsids are hypersensitive to elevated temperature and dissociated in this condition (Doel and Baccarini 1981). Therefore, our results suggest that the yeast-expressed polyprotein is appropriately processed and subsequently assembled into VLPs, and is dissembled by boiling.

**Fig. 4:**
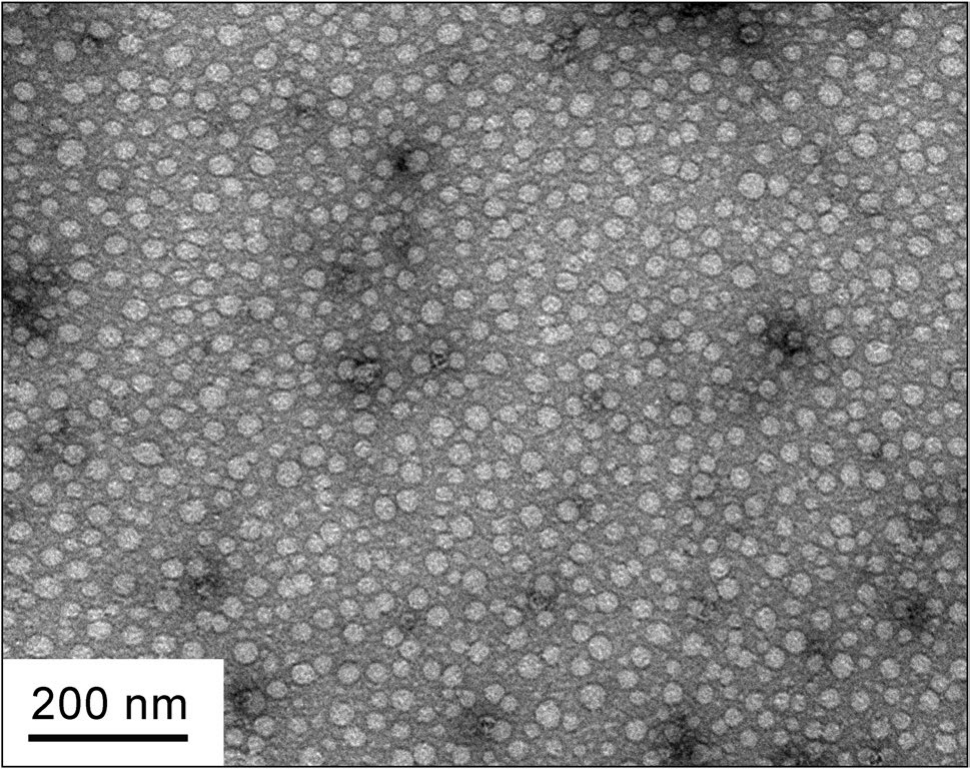
Electron microscopy image of a purified virus particle. Virus particles approximately 30-nm diameter are shown as an icosahedral structure under EM after negative staining with 2% uranyl acetate

EM of the partially purified viral preparation confirmed the existence of empty isometric particles of size at around 30 nm in diameter that showed the characteristic black spots associated with heavy-metal stain in their interior (Fig. 4). These were similar in size to the FMDV viral particle (Dong et al. 2021; Fry et al. 1999; Jamal and Belsham 2013). These results indicate that in yeast, *S. cerevisiae*-expressed P1 precursor polyprotein is processed into capsid subunit proteins by co-translated 3C protease and then subsequently assembled into a virion particle.

VLP expression levels were indirectly measured by comparing the band intensity of the serially diluted-known amount of *E. coli*-expressed VP3. As shown in Supplemental Fig. S3, the band intensity of the processed VP3 from the yeast-expressed VLPs fell approximately between 0.5 and 1.0 μg, which is equal to 0.5% of protein preparation, from which we infer that VLPs equivalent to the amount of 15 mg of VP3 were produced by a liter culture of recombinant yeast. In addition, VLP amount was measured by comparison to the known amount of FMDV using the commercially available detection kit. Consistent with previous results using VP3 subunit, this result suggested that approximately 50 mg of VLPs per liter of culture was produced.

### Immune response of expressed VLPs

**Fig. 5:**
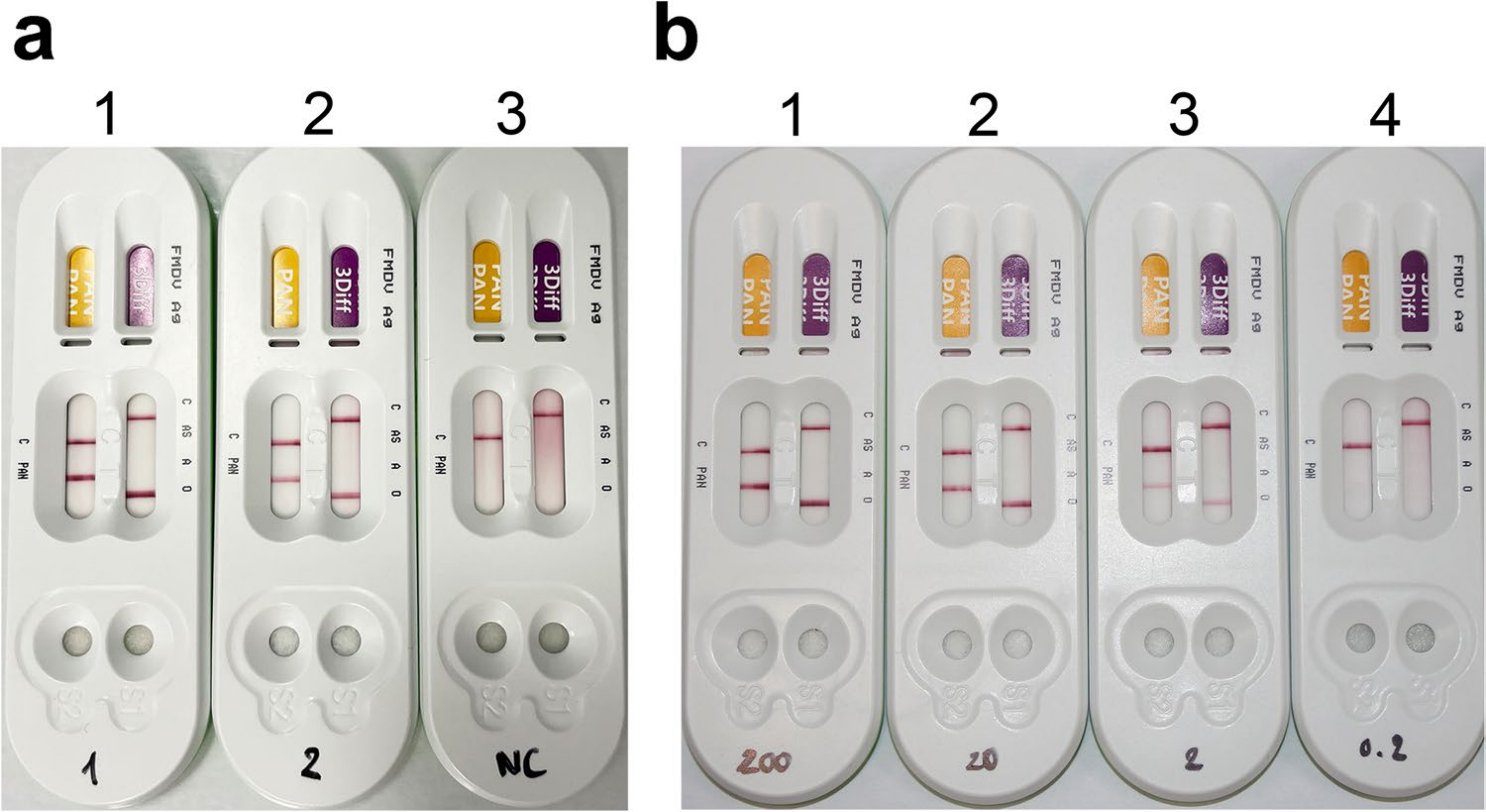
FMDV detection using a commercially available FMDV diagnosis kit. **a** Preparation of total soluble protein from transformant (1) and partially purified VLPs (2) after the ultracentrifugation were tested using the kits. The left two bands indicate FMDV-positive while the right two bands specify serotype O. Note that only a single band for each was detected by the sample prepared from the mock-transformant used as a negative control (3). **b** Serial dilution of total soluble protein from recombinant yeast was tested to identify the lowest concentration sufficient to be detected by the kit. Testers 1, 2, 3, and 4 contain 2 mg, 200 μg, 20 μg, and 2 μg of total soluble protein, respectively

The antigenicity of the yeast-expressed VLPs was tested using a commercially available FMDV antigen detection kit (VDRG® FMDV 3Diff/PAN Ag Rapid Kit, Median Diagnostics Inc.). An antigen–antibody reaction was detected in protein preparation from both the recombinant yeast as well as those from the partially purified-VLPs. Samples of as low as 20 μg of protein prepared from the recombinant yeast were detectable by the kit (Fig. 5). These results suggest that the antigenicity of the VLPs was well maintained through preservation of native FMDV particle integrity. Therefore, the VLPs are thought to be anti-genically strong enough to induce an immune response and protect further infection of FMDV.

## Discussion

We demonstrated a high expression level of FMDV structural proteins and the proteolytic process associated with the structural polyprotein of FMDV using *S. cerevisiae*. The presence of discrete 3C protein suggested that the 2A peptide operated in the company of the N-terminal serine residue of the 3C protein to modify the translational machinery, thereby releasing nascent P1-2A polyprotein from the ribosome while allowing re-initiation of the translation of the downstream 3C protein via a novel translational effect (de Felipe et al. 2003; Donnelly et al. 2001; Sharma et al. 2012). It therefore became possible to produce a co-translated but discrete 3C protein from a monocistronic transcript, which proved that the 2A protein with an N-terminal serine of 3C achieved a novel ribosomal skipping translation mechanism in *S. cerevisiae* that released the nascent polyprotein and re-initiated the translational mechanism of the 3C protease from a monocistronic transcript. In addition, substituting proline with serine at the 2A/3C junction (Fig. 1), which has been reported to reduce cleavage efficacy to a level lower than the authentic 2A/2B junction (Kjær and Belsham 2018), operated as expected, resulting in only a small amount of discrete 3C protein (the consequence of lowered ribosomal skipping efficacy). This lowered production of discrete 3C protein was evidenced by the weak intensity of the cross-reacting band corresponding to the 3C protein in the western analysis (Fig. 2d). Considering the harmful effect of 3C protease on host cells, the reduced expression of 3C protein might be considered beneficial for yeast cell growth, a result that is consistent with the results of the normal cell growth of transformants.

The presence of discrete bands of VP0, VP3, VP1, and 3C clearly indicated that the P1-2A-3C gene was translated to produce a polyprotein P1 which is then proteolytically cleaved into capsid proteins of VP0, VP3, and VP1 by co-translated 3C protease in yeast. Interestingly, the unequal intensity of the bands corresponding to viral subunits, specifically the very faint band intensity of the VP1 subunit, was unexpected based on the equimolar production of viral subunits due to the proteolytic processing by 3C protein from a polyprotein P1. Although further studies will be required in light of the weak response on the western analysis, our in situ analysis of *O*- and *N*-glycosylation of our protein product of VP1 using NetOGly 3.1 (http://www.cbs.dtu.dk/services/NetOGlyc/) and NetNGlyc-1.0 (https://services.healthtech.dtu.dk/services/NetNGlyc-1.0/) revealed the presence of six predicted *O*-glycosylation and two predicted *N*-glycosylation sites within VP1. These results may explain the poor cross-reaction of yeast-derived VP1 against the antibodies raised against *E. coli*-expressed VP1 protein. Western blot analysis using the boiled protein samples of five resulting transformants showed the presence of an uncleaved polyprotein around 100 kDa regardless whether anti-VP1, -VP2, or -VP3 antibodies and were used (Supplemental Fig. S4), indicating that the protein product of the P1 gene is expressed as a single polyprotein without further processing. In our western analysis, it is interesting to observe a band which corresponded to the size of VP2 only in addition to the VP0 band because the VP0 processing, also known as maturation cleavage, is accompanied by capsid assembly with the packaging of genomic RNA. Thus, the discrete VP2 band indicated an incomplete cleavage of VP0 and capsid assembly even devoid of RNA genome, which has been observed before in VLP production using a recombinant baculovirus (Subramanian et al. 2012).

In conclusion, we proved that the 2A protein with an N-terminal serine of 3C achieved a novel “ribosomal skipping” translation effect in *S. cerevisiae*, which results in the release of the nascent polyprotein and re-initiation of translation of the 3C protease from a monocistronic transcript. The resulting discrete 3C protease then proteolytically processes the structural polyprotein into capsid proteins VP0, VP1, and VP3. More importantly, examination of the transformants by electron microscopy showed the presence of the characteristic icosahedral virus-like particles (VLPs). Our results clearly demonstrate that *S. cerevisiae* processes the viral structural polyprotein using a viral 3C protease, which is mediated by the 2A sequence for self-processing, and the resulting viral capsid subunits are assembled into VLPs.

## Supplementary Information

Below is the link to the electronic supplementary material.

Supplementary file1 (PDF 1297 KB)

## Data Availability

All the data supporting the findings of this study are available within the paper and its supplementary information.
