# Supplementary material for: Expression of virus-like particles (VLPs) of foot-and-mouth disease virus (FMDV) using Saccharomyces cerevisiae
Source: Appl Microbiol Biotechnol. 2024 Jan 9;108:81. doi: 10.1007/s00253-023-12902-9 (PMC10776484; doi:10.1007/s00253-023-12902-9)
Supplement: Supplementary file 1 [file 253_2023_12902_MOESM1_ESM.pdf]

Supplementary Information

Journal Name: Applied Microbiology and Biotechnology

Article Title: Expression of virus-like particles (VLPs) of foot-and-mouth disease virus (FMDV) using *Saccharomyces cerevisiae*

Author name and Affiliations: Ngoc My Tieu Le[1] · Kum-Kang So[2] · Jeesun Chun[2] · Dae-Hyuk Kim[1,2]

[1]Department of Bioactive Material Sciences, Jeonbuk National University, Jeonju, Jeollabuk-do, Republic of Korea

[2]Institute for Molecular Biology and Genetics, Department of Molecular Biology, Jeonbuk National University, Jeonju, Jeollabuk-do, Republic of Korea

Ngoc My Tieu Le, Kum-Kang So and Jeesun Chun contributed equally to this work and share first authorship.

*** Correspondence**

Dae-Hyuk Kim, Department of Bioactive Material Sciences, Institute for Molecular Biology and Genetics, Department of Molecular Biology, Jeonbuk National University, Jeonju, 54896, Republic of Korea.

E-mail: dhkim@jbnu.ac.kr

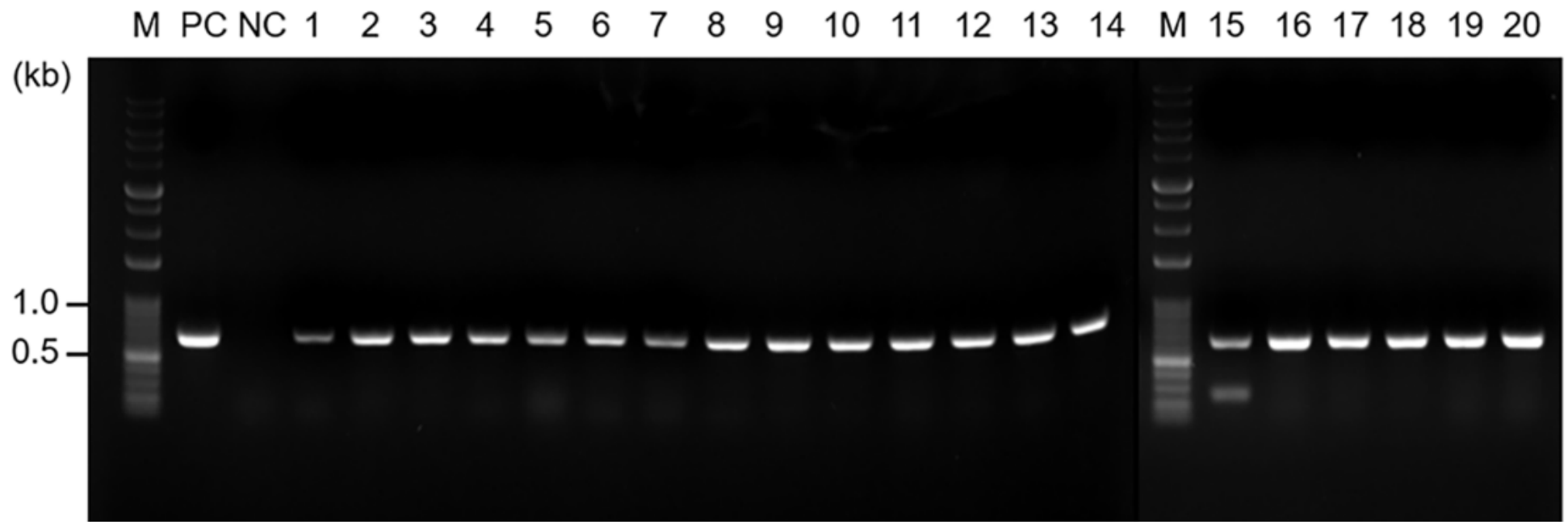

Fig. S1 Colony PCR of yeast transformants. Agarose gel electrophoresis was conducted to show the expected 640-bp PCR amplicons using primers for amplification of the 3C sequence. Lanes 1-20 contain the products of the colony PCR using putative transformants as templates. PC contains the PCR product from the reaction using pYEGPD-P1-2A-3C as a template and NC contains the PCR products using mock (vector only) transformant. Numbers on the left indicate the size in kb

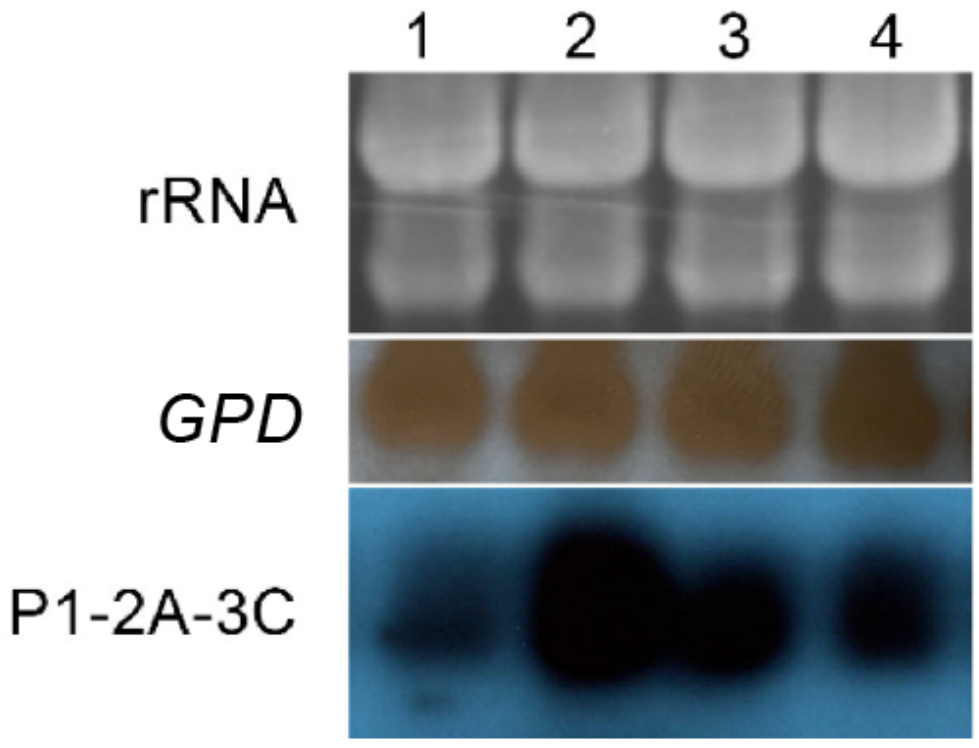

Fig. S2 Analysis of temporal expression of the P1-2A-3C transcript from representative transformant TpYEGPD-P1-2A-3C-3. Lanes 1-4 contain 30 μg each of total RNA from the 1-, 2-, 3- and 5-day cultures of the selected transformant, respectively. The rRNA is shown to indicate an equal loading amount of RNA and *GPD* transcription is shown as an internal control

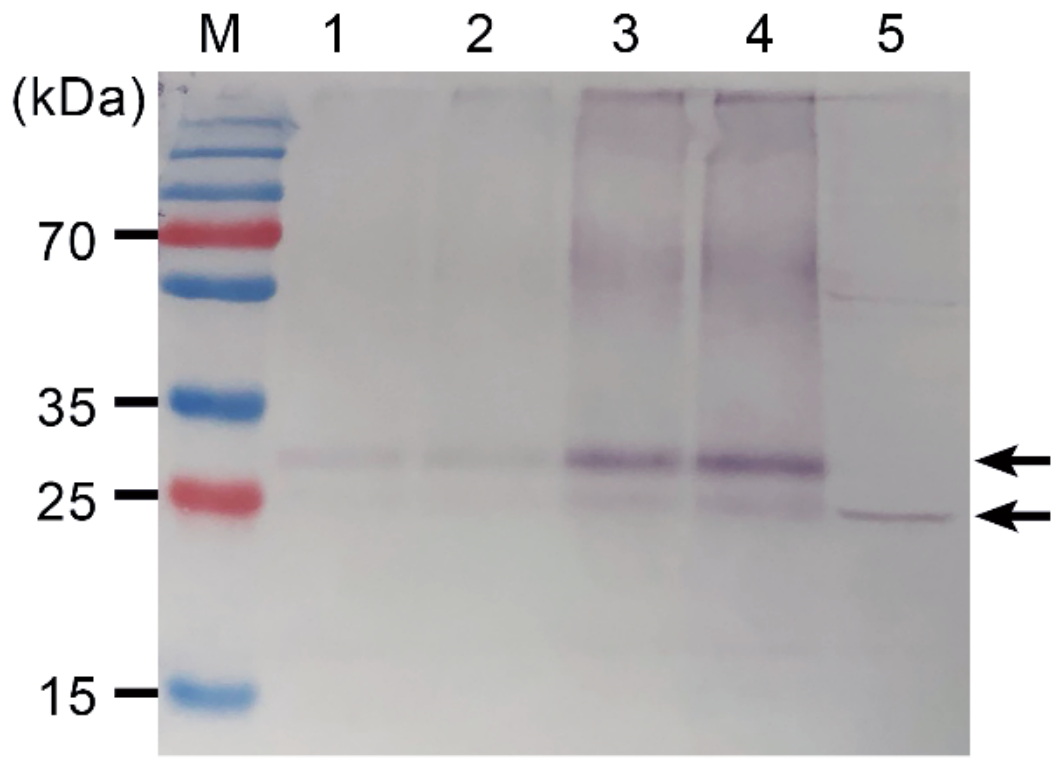

Fig. S3 Western blot analysis of yeast expressing P1-2A-3C polyprotein to measure VLP. Western blot analysis of serially diluted-known amount of *E. coli*-expressed VP3 was compared with those of yeast expressing VP3. Lanes 1-4 contain 0.5, 1.0, 2.0, and 3.0 μg of *E. coli*-expressed VP3 protein. Densitometry of cross-reacting band intensity was performed to estimate the protein amount

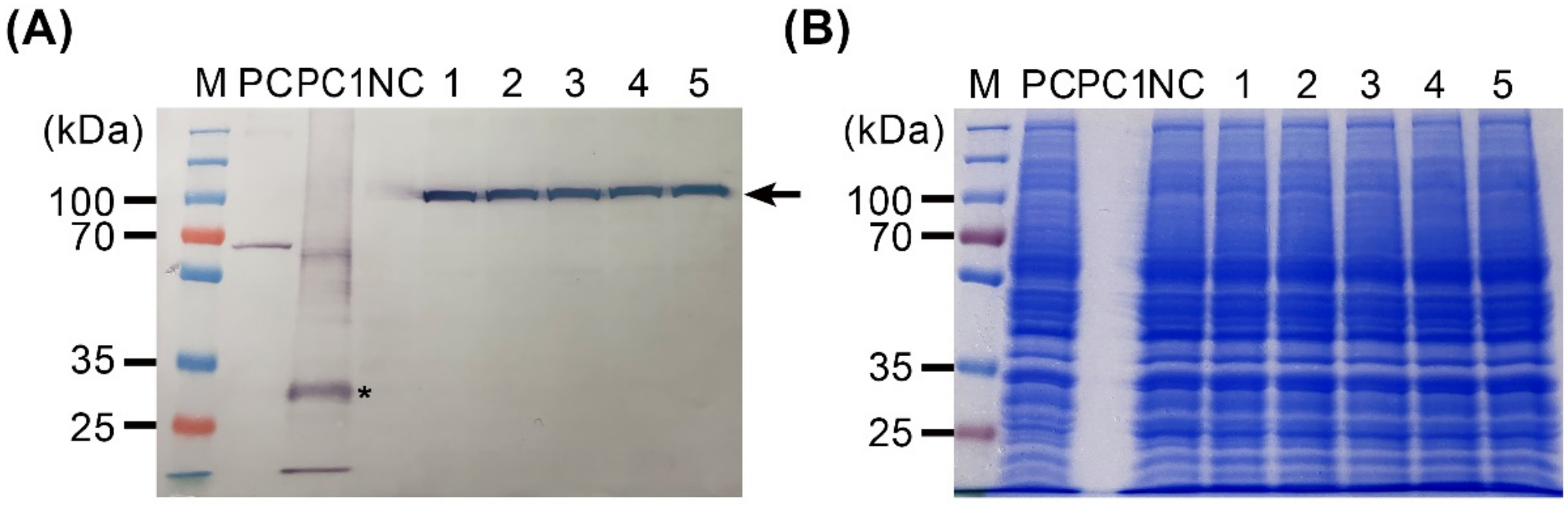

Fig. S4 Expression analysis of yeast codon optimized P1 polyprotein. (A) Western blot analysis of yeast expressing P1 polyprotein using an antibody against VP3. PC: VP0-VP3 fusion of protein from yeast, PC1: VP3 protein from *E. coli*. NC: protein preparation from mock (vector only) transformant. Lanes 1-5 contain protein preparations from selected five yeast transformants cultured for 3 days at 30 ºC. (B) A representative twin SDS-PAGE gel stained with Coomassie blue is shown to confirm an equal loading amount

Table S1 List of primers used in this study

Primer	Sequence (5′ to 3′)	Applications
*Bam*HI-VP4-F	GGATCC**ATG**GGAGCCGGTCAATCTAGCCCG	Cloning P1 and P1-2A-3C into pYEGPD-TER
*Bam*HI-VP2-F	GGATCCGACAAGAAAACCGAAGAG	Cloning VP2 into pQE-30
*Sal*I-VP2-R	GTCGACTTCCTTGGAAGGGAATTCACC	Cloning VP2 into pQE-30
*Sal*I-TAA-VP1-R	GTCGAC**TTA**TTGTTCCACTGGTGCAAC	Cloning P1 into pYEGPD-TER
*Bam*HI-3C-F	GGATCCTCTGGTGCACCACCGACT	Cloning 3C into pQE-30
*Sal*I-TAA-3C-R	GTCGAC**TTA**TTCATGGTGTGGTTCA	Cloning P1-2A-3C into pYEGPD-TER
*Sal*I-3C-R	GTCGACTTCATGGTGTGGTTCA	Cloning 3C into pQE-30
*Bam*HI-VP1-F	GGATCCACTACCTCCGCCGGCG	Cloning VP1 into pColdII
*Sal*I-VP1-R	GTCGAC TTGTTCCACTGGTGCAAC	Cloning VP1 into pColdII
*Bam*HI-VP3-F	GGATCCGGGATCTTTCCAGTGGC	Cloning VP3 into pQE-30
*Sal*I-VP3-R	GTCGACCTGTGTTCTAGCATCC	Cloning VP3 into pQE-30
GPD probe F	GTTTCCCATGACGACAAGCACA	Probe for Northern blot analysis
GPD probe R	CAACTTGACGAACTTTGGAGACAAT	Probe for Northern blot analysis

Underlines indicate recognizing sequences for corresponding restriction enzymes.

Bolds indicate the start and stop codon sequences.
